# Cell Immobilization Using Alginate-Based Beads as a Protective Technique against Stressful Conditions of Hydrolysates for 2G Ethanol Production

**DOI:** 10.3390/polym14122400

**Published:** 2022-06-14

**Authors:** Raiane C. Soares, Teresa C. Zangirolami, Raquel L. C. Giordano, Mekonnen M. Demeke, Johan M. Thevelein, Thais S. Milessi

**Affiliations:** 1 Institute of Natural Resources, Federal University of Itajubá, Av. Benedito Pereira dos Santos, 1303, Itajubá 37500-903, MG, Brazil; raiane.soares@unifei.edu.br; 2 Program of Chemical Engineering, Federal University of São Carlos, Rodovia Washington Luíz, km 235, São Carlos 13565-905, SP, Brazil; teresacz@ufscar.br (T.C.Z.); raquel@ufscar.br (R.L.C.G.); 3 NovelYeast bv, Open Bio-Incubator, Erasmus High School, Laarbeeklaan 121, B-1090 Brussels, Belgium; mekonnen.demeke@novelyeast.com (M.M.D.); johan.thevelein@novelyeast.com (J.M.T.)

**Keywords:** cell immobilization, alginate hybrid gel, recombinant yeast, 2G ethanol, hydrolysate inhibitors

## Abstract

The development of biorefineries brings the necessity of an efficient consumption of all sugars released from biomasses, including xylose. In addition, the presence of inhibitors in biomass hydrolysates is one of the main challenges in bioprocess feasibility. In this study, the application of Ca-alginate hybrid gels in the immobilization of xylose-consuming recombinant yeast was explored with the aim of improving the tolerance of inhibitors. The recombinant yeast *Saccharomyces cerevisiae* GSE16-T18SI.1 (T18) was immobilized in Ca-alginate and Ca-alginate–chitosan hybrid beads, and its performance on xylose fermentation was evaluated in terms of tolerance to different acetic acid concentrations (0–12 g/L) and repeated batches of crude sugarcane bagasse hemicellulose hydrolysate. The use of the hybrid gel improved yeast performance in the presence of 12 g/L of acetic acid, achieving 1.13 g/L/h of productivity and reaching 75% of the theoretical ethanol yield, with an improvement of 32% in the xylose consumption rate (1:1 V_beads_/V_medium_, 35 °C, 150 rpm and pH 5.2). The use of hybrid alginate–chitosan gel also led to better yeast performance at crude hydrolysate, yielding one more batch than the pure-alginate beads. These results demonstrate the potential of a hybrid gel as an approach that could increase 2G ethanol productivity and allow cell recycling for a longer period.

## 1. Introduction

The search for sustainable global development and the transition of the energy matrix towards non-fossil sources have led to biorefinery designs which require excellence and competitiveness associated with the fulfillment of basic performance requirements: use of the entire biomass, minimal waste generation, and fostering sustainable socioeconomic development [1,2]. In addition, the viability of transport biofuels also depends on other factors, such as net energy gain, commercial competitiveness, large-scale producibility, and environmental benefits [3]. In this context, bioethanol stands out as a potential biofuel, since it is already produced on a global scale.

First-generation bioethanol (1G) production is based on a traditional and well-stablished worldwide technology, which uses fermentable sugars directly extractable from food cultures (saccharide or starchy), employing efficient industrial native *Saccharomyces cerevisiae* yeast strains. Second-generation bioethanol (2G), on the other hand, is produced from lignocellulosic biomass and, besides increasing biorefinery sustainability, it also does not compete with food production. However, some obstacles are still present in 2G ethanol production, due to low conversion efficiency and yield, difficulty of fermentable sugar release, implementation of efficient biomass pretreatments, and the need for efficient fermentation of all sugars released from biomass [4,5].

Lignocellulosic raw materials, such as sugarcane bagasse and straw, are mainly composed of cellulose, hemicellulose, and lignin, presenting a recalcitrant matrix that needs to be disrupted to make the fermentable sugars from hemicellulose and cellulose available. In order to circumvent this complex structure, a pretreatment step is mandatory [6], releasing mainly glucose from cellulose and xylose from hemicellulose fractions. The ethanol production from C6 sugars can be done in the well-established 1G process using *S. cerevisiae*. In addition, within the biorefinery concept, many technological solutions for cellulose fraction have been proposed to produce other valuable chemicals, and some of them have been developed at an industrial scale, such as biopolymer production by Braskem [7]. However, the use of the hemicellulose fraction from biomass is a challenge; this fraction still is industrially underused.

Non-GMO *S. cerevisiae* strains show poor fermentation of C5 sugars due to the absence of endogenous enzymes [5]. Naturally C5-fermenting yeasts, on the other hand, generally demand a micro-aerated environment and exhibit a low xylose fermentation rate and productivity [8], which makes its application at an industrial scale difficult. To overcome this challenge, it is necessary to develop strains capable of fermenting xylose efficiently, which will increase ethanol production from biomass without increasing the cultivation area [9]. In this sense, with advances in molecular biology and metabolic engineering, recombinant strains with superior C5 sugar assimilation have been developed [10,11,12]. This is an essential requirement to circumvent the underutilization of the hemicellulose biomass fraction and a prerequisite for feasible bioeconomic production, so that bioethanol generation can also become based on xylose.

Besides the potential use of recombinant yeasts, attention has to be given to the high concentration of inhibitors present in lignocellulosic hydrolysates, which are grouped into three main categories: furan-aldehydes, aliphatic acids (such as acetic acid), and phenolic compounds [13]. These inhibitors are produced during pretreatment steps and can significantly jeopardize the fermentation performance, especially for some recombinant yeasts. In addition, the inserted capacity to ferment C5 sugars could lead to a higher sensibility to these compounds [10,14]. On the other hand, the inclusion of detoxification steps to reduce the concentration of inhibitory compounds significantly increases operation time and process costs [15].

An alternative to overcome the aforementioned adversities is cell immobilization on solid supports, which provides a microenvironment inside gel beads to protect the yeasts strains against harmful agents present in the hydrolysates [16]. This technique has other advantages, such as the easy recovery and reuse of the biocatalyst in repeated batches, the operation of the bioreactor with high cell densities for long periods, and the reduction in biomass management (production, transport, and storage) [17]. In addition, once the cells are confined into a solid support, it helps the insertion of genetically modified organisms (GMO) in industrial processes for countries with strict biosafety regulations [15,18]. Furthermore, cell immobilization by entrapment can be carried out with natural polymers, such as alginate and chitosan, which are non-toxic, biocompatible, and biodegradable.

Sodium alginate is the most studied natural polymer for the immobilization of viable cells. It is a linear polysaccharide present in brown algae consisting of mannuronic acid units linked by β-(1,4) glycosidic bonds and also by guluronic acid units, joined by α-(1,4) bonds [19,20]. The main advantage of using this material is related to its rapid gelation under mild conditions, which allows the preservation of the activity and viability of the immobilized microorganisms [21]. Chitosan is a low-cost cationic support, which has amino groups arranged in its structure and is produced by deacetylation of chitin, a natural polymer present in the cell wall of fungi and mollusk shells [22]. Furthermore, both are polyelectrolytes with a wide range of laboratory and industrial applications, such as thickening agents, controlled release system, healing biomaterials, cell culture, ion exchange material for the removal of toxic compounds, etc. The potential of alginate to adsorb several biological and toxic compounds is well described in the literature [23,24,25]. When alginate and chitosan are used together, the formation of a polyelectrolyte complex is observed, receiving attention due to its high biocompatibility and potential in the development of tenacious biomaterials [26,27].

In this context, the present work aimed to evaluate the use of alginate–chitosan hybrid gels as a protective shield against hydrolysate inhibitors with the aim of improving the production of 2G ethanol from crude sugarcane bagasse hemicellulose hydrolysates by the recombinant yeast *Saccharomyces cerevisiae* GSE16-T18SI.1 (T18). The use of hybrid alginate gels for this purpose is not explored much in the literature. In the present work, the performance of a cell entrapped in a hybrid alginate–chitosan gel is compared with pure alginate beads, and it is shown that the hybrid gel is a potential alternative, increasing 2G ethanol productivity and allowing cell recycling for a longer period.

## 2. Materials and Methods

### 2.1. Materials

Sodium alginate was purchased from Sigma-Aldrich (St. Louis, MO, USA). Chitosan (85% deacetylation) from Polymar (Fortaleza, CE, Brazil). Sodium borohydride was from Vetec (São Paulo, SP, Brazil). All other chemicals were of analytical grade and were used without any treatment.

### 2.2. Microorganism and Inoculum

The recombinant yeast *Saccharomyces cerevisiae* GSE16-T18SI.1 (T18) was used in all experiments. This strain is engineered for xylose fermentation by the insertion of multiple copies of the *Clostridium phytofermentans* xylose isomerase and subsequent genome shuffling techniques [11]. The biomass production for cell immobilization was performed in YPXD 2% medium (10 g/L yeast extract, 20 g/L peptone, 10 g/L xylose, 10 g/L glucose; sterilized at 121 °C for 20 min) supplemented with 100 μg/mL of ampicillin [15]. Firstly, a pre-inoculum was prepared by adding a loop of the stock culture to 3 mL of YPXD 2%. After incubation for 12 h at 30 °C and 200 rpm, 10 mL of cells were poured into 250 mL of YPXD 2% in a 1 L flask and the inoculum was kept at 30 °C and 200 rpm for 24 h. For immobilization, the yeast cells were recovered in the exponential phase by centrifugation (4500 rpm for 30 min).

### 2.3. Preparation of Chitosan Gel

Chitosan gel was prepared through the methodology of Silva et al. [28], where the coagulation of the chitosan-acetic acid solution (2%, *w*/*v*) is performed in 0.5 M KOH (60 min, 300 rpm, 50 °C) followed by the activation with glutaraldehyde (0.8%, *v*/*v*) at pH 7.0 (100 mM phosphate buffer, 1:10 m_gel_/v_suspension_). After 60 min stirring at 25 °C, the chitosan gel was filtered and extensively washed with ultrapure water until neutrality.

### 2.4. Yeast Encapsulation in Different Alginate-Based Beads

T18 yeast immobilization in different alginate-based beads was performed aseptically in a biological safety cabinet by encapsulation methodology adapted from Milessi et al. [15]. A suspension of 10% (*w*/*w*) of yeast cells (100% cell viability) in 1% (*w*/*w*) of sodium alginate was dropped into a coagulation solution (CaCl_2_ 0.25 M and MgCl_2_ 0.25 M) to produce the pure Ca-alginate beads. Yeast concentration was determined as described in Item 2.7.2 to ensure the same cell concentration in both types of alginate beads. The hybrid Ca-alginate–chitosan beads were obtained by dropping a suspension containing 1% (*w*/*w*) of sodium alginate, 15% (*w*/*w*) of chitosan gel, and 10% (*w*/*w*) of yeast cells (100% cell viability) into the same coagulation solution. In both procedures, beads of small diameter (1–3 mm) were obtained using a pneumatic extruder, adapted from Trovati et al. [29]. Due to the high content of water in the Ca-alginate and Ca-alginate/chitosan (85% of moisture), the immobilized biocatalysts were maintained for 12 h at 4 °C in a cure solution composed of fermentation medium without the carbon source to avoid changes in pH and medium composition when added to the fermentation experiments.

### 2.5. Acetic Acid Tolerance Experiments

All fermentation experiments were carried out in triplicate at 35 °C as previous reported [11,15], using flasks with 8 mL of reaction volume containing free or immobilized cells (50 g/L; OD_0_ = 100). Small-scale experiments are a reliable and efficient technique that has been widely used for several purposes [30]. The reliability of the experiments carried out in 8 mL mini-reactors was previously assessed and validated for bioethanol production, reproducing the fixed-bed reactor results for hemicellulose hydrolysate fermentation [15,31]. Thus, this experimental strategy saves time and experimental material and enables the collection of reliable data at the laboratory scale, which generates a large amount of experimental data using a simple monitoring procedure and preserving fermentative conditions. The experiments to access acetic acid tolerance were performed on YPX 40 g/L of xylose and pH 5.2, containing different concentrations of acetic acid (0 to 12 g/L) at 150 rpm, 35 °C, and initial pH 5.2. The medium was prepared with double the desired initial concentrations of xylose and acetic acid due to the dilution caused by the addition of beads. Fermentations were monitored by measuring weight loss due to CO_2_ release, as described in Item 2.8 [5].

### 2.6. Repeated Batch Experiments

Repeated batch experiments were performed using sugarcane bagasse hemicellulose crude hydrolysate (without detoxification) with 50 g/L of immobilized cells (OD_0_ = 100). The hydrolysate was not sterilized; it was supplemented with 2.0 g/L of urea and 100 μg/mL of ampicillin (to prevent contamination). The medium pH was adjusted to 5.2 with Ca(OH)_2_, followed by filtration to remove suspended solids. All fermentations were carried out at 150 rpm, 35 °C, and initial pH 5.2 and monitored by measuring weight loss due to CO_2_ release, as described in Section 2.5. The end of a batch was defined by the cessation of the CO_2_ release. A new batch was started by adding new fermentation medium after the removal of the fermented one, without washing the cell beads between batches since the strategy of washing the encapsulated cells in between batches does not improve yeast performance in ethanol production, as reported by Wang et al. [32].

### 2.7. Analytical Methods

#### 2.7.1. Substrate and Product Quantification

High-performance liquid chromatography (HPLC) analysis was performed for the quantification of substrates (glucose and xylose), products (ethanol, xylitol, glycerol), and inhibitors (acetic acid, furfural, hydroxymethylfurfural-HMF) The chromatograph Model 10AD (Shimadzu, Kyoto, Japan) was equipped with refractive index and UV–visible detectors. Analyses of xylose, ethanol, xylitol, glycerol, and acetic acid were performed in an Aminex HPX87-H column at 45 °C with H_2_SO_4_ (5 mM) as eluent (0.6 mL/min). Before injections, hydrolysate samples were filtered in Sep-Pak^®^ C-18 (Waters, Milford, MA, USA) cartridges. Furfural and HMF were quantified using a C-18 (Beckman) column connected to a UV–visible detector (274 nm) using as eluent 0.8 mL/min acetonitrile/water 1:8 with 1% (*v*/*v*) acetic acid.

#### 2.7.2. Cell Concentration and Viability

Cell concentration was determined by turbidimetry using a spectrophotometer (Ultrospec 2100 pro (Biochrom, Holliston, MA, USA)), correlating the measured optical density (OD) at 600 nm with cell dry weight (g/L) through a previously determined calibration curve. Cell viability was quantified using methylene blue staining technique [33], followed by counting viable cells in a Neubauer’s chamber. To release the immobilized cells for viability assays, the biocatalyst beads were suspended in 8% (*w*/*v*) sodium citrate buffer (100 mg_beads_/mL) under magnetic stirring to solubilize the alginate gel [15]. Cell viability was defined as the ratio between viable cells and total cells counted in a defined space of the chamber.

### 2.8. Calculations

The final performance indexes, substrate conversion (%), overall ethanol yield Y_P/S_ (g_ethanol_/g_substrate_), and volumetric productivity Q_P_ (g/L/h) were calculated according to Shuler and Kargi [34] using Equations (1)–(3) as follows, where C_Sf_—final substrate concentration (g/L), C_S0_—initial substrate concentration (g/L), C_Pf_—final ethanol concentration (g/L), C_P0_—initial ethanol concentration (g/L), and t_f_—total time of fermentation (h). Initial and final substrate and ethanol concentrations were assessed by HPLC analyses. For estimation of Y_P/S_ and Q_P_ in the repeated batch experiments, only the produced ethanol during a batch was considered by discounting the ethanol concentration retained inside the beads from the previous batch (measured in the beginning of a new batch through HPLC analysis).

Conversion = [(C_Sf_ − C_S0_) × 100]/C_S0_ (1)

Y_P/S_ = (C_Pf_ − C_P0_)/(C_S0_ − C_Sf_) (2)

Q_P_ = (C_Pf_ − C_P0_)/t_f_ (3)

The xylose consumption rate (r_S_, g/L/h) was calculated according to Equation (4) [34], adjusting a polynomial in the xylose consumption over time curve and extracting the tangent of the curve (dC_S_/dt) through derivation of the polynomial. Statistical analysis of data was performed through a Tukey test with a 95% confidence level using the software OriginPro 9 (Originlab, Northampton, MA, USA).

r_S_ = −dC_S_/dt (4)

The comparison between xylose consumption rate using the two gels was calculated according to Equation (5), taking into account the increase in consumption rate using Ca-alginate/chitosan beads (r_SAC_) compared with the consumption rate using Ca-alginate beads (r_SA_), expressed as a percentage value of improvement (%).

Increase on xylose consumption = [(r_SAC_ − r_SA_) × 100]/r_SA_ (5)

### 2.9. Scanning Electron Microscopy (SEM) of Alginate-Based Beads

Scanning electron microscopy (SEM, XL30-FEG, FEI) of the different alginate gel beads was performed to qualitatively compare the alginate-based beads. The polymers containing immobilized cells were freeze dried, and samples were coated with Au-Pd using a sputtering device (Edwards S150 (Edwards Vacuum, Burgess Hill, UK)) to produce a thin conductive film on the surface. SEM was operated at standard high-vacuum settings, using 10 mm of working distance and 10.0 keV of accelerating voltage [16].

## 3. Results

### 3.1. Acetic Acid Tolerance of T18 Yeast Encapsulated in Different Alginate-Based Beads

**Figure 1:**
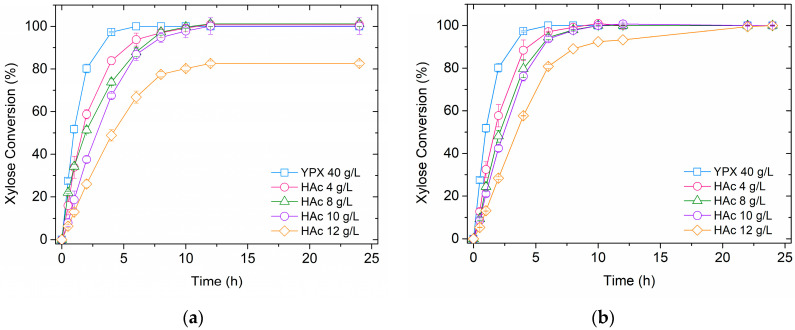
Fermentation performance of immobilized T18 in YPX (40 g/L) in different acetic acid (Hac) concentrations. (**a**) Ca-alginate beads and (**b**) Ca-alginate/chitosan beads. Experiments performed with 1:1 V_beads_/V_medium_, 35 °C, 150 rpm, and pH 5.2.

Acetic acid (HAc) is a well-known inhibitor of *S. cerevisiae*, and its production during biomass pretreatment for 2G ethanol production is inevitable due to deacetylation of the hemicellulose fraction [12]. It strongly affects T18 free cell metabolism, reducing the xylose fermentation rate by 10-fold in the presence of 8 g/L of acetic acid [15]. The potential of alginate gel beads in the protection of yeasts cells has been shown previously [15,16]; however, there is still room for improvement, and the application of hybrid polymers could be an interesting approach. In this sense, to assess the yeast protection against toxic compounds provided by the polymers used in immobilization process, the performance of the yeast T18 immobilized in Ca-alginate beads and in hybrid Ca-alginate/chitosan beads was evaluated during xylose fermentations with different initial concentrations of acetic acid (from 0 to 12.0 g/L). The conversion profiles for different fermentation conditions are shown in Figure 1. Table 1 gathers the main performance indexes for each studied condition.

**Figure 2:**
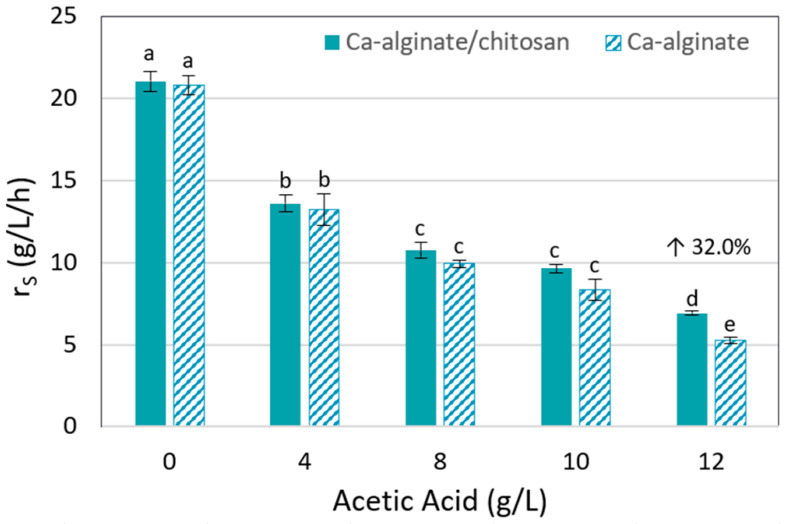
Xylose consumption rate (r_S_) in YPX (40 g/L) with different acetic acid concentrations by T18 yeast immobilized in Ca-alginate and Ca-alginate/chitosan gel. Experiments performed with 1:1 V_beads_/V_medium_, 35 °C, 150 rpm, and pH 5.2. Increase in xylose consumption represented as percentage (up arrow). (a–e) Matching letters on top of the bars indicate that the means did not differ significantly, according to the Tukey test with 95% confidence level.

The strong influence of acetic acid on T18 metabolism is clear, with ethanol productivity significantly decreasing with increasing acid concentration. It is also interesting to observe that the ethanol yield in the presence of acid was inferior to without it, probably due to the deviation of substrate and cell energy to the cell maintenance under stressful conditions. T18 yeast consumed xylose efficiently in all experiments, except when Ca-alginate beads were used in the presence of 12 g/L of acetic acid, where 6.4 g/L of residual xylose was observed. The use of the hybrid gel of Ca-alginate/chitosan improved the yeast tolerance to this inhibitor, fermenting all xylose in the medium containing 12 g/L of acetic acid, with 1.13 g/L/h of productivity and achieving 75% of ethanol theoretical yield. It is worth highlighting that in all experiments, the formation of byproducts (such as xylitol) was not observed, and final cell viability remained above 90%. To better visualize the hybrid polymer influence on yeast performance, the substrate consumption rate (r_S_) of each studied condition was determined, as presented in Figure 2. It is clear that improvement was provided by the hybrid polymer in the presence of 12 g/L of acid, reaching an r_S_ 32%, higher than with the Ca-alginate beads. These results indicate that the application of Ca-alginate/chitosan beads in the T18 yeast encapsulation can improve its performance on 2G ethanol production from biomass hydrolysates. Repeated batch experiments using sugarcane bagasse hemicellulose hydrolysate were performed to assess this hypothesis.

### 3.2. Repeated Batch Experiments in Crude Sugarcane Bagasse Hemicellulose Hydrolysate

**Figure 3:**
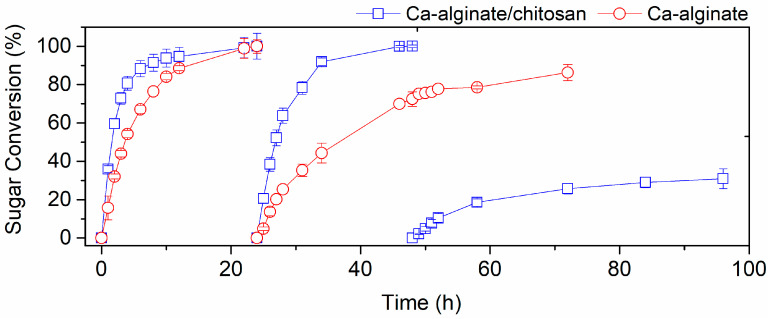
Recycling for sugarcane bagasse hemicellulose hydrolysate supplemented with urea (2 g/L) using T18 yeast immobilized in Ca-alginate (red circles) and in Ca-alginate/chitosan hybrid gel (blue squares). Experiments performed with 1:1 V_beads_/V_medium_, 35 °C, 150 rpm, and pH 5.2.

Due to the interesting improvement on yeast performance in the presence of acetic acid, the T18 yeast encapsulated in both gels was applied in the production of 2G ethanol in repeated batches using crude sugarcane bagasse hemicellulose hydrolysates. Besides the protection provided by the encapsulation gel, the ease of reuse is a great advantage of immobilized cells and could favor its industrial application. The hydrolysate used in these experiments was kindly donated by Praj Industries (India). It was composed of 16.7 g/L of glucose, 85.7 g/L of xylose, 7.8 g/L of arabinose, 7.7 g/L of acetic acid, 0.29 g/L of furfural, and 0.67 g/L of hydroxymethyl furfural and was supplemented with 2 g/L of urea. This hydrolysate showed to be very toxic for T18 free cells, which were not able to grow in this medium; all cells were dead after 24 h of process. Encapsulated T18, on the other hand, was capable to perform repeated batches on crude hydrolysate due to the polymer’s protection. As shown in Figure 3 and Table 2, for Ca-alginate beads, the yeast’s performance and viability was considerably reduced in the second recycle, decreasing ethanol productivity to less than half, with incomplete sugar consumption (7.8 g/L of residual sugars). The process improvement using hybrid Ca-alginate/chitosan beads is clear (Figure 3 and Table 2). Xylose consumption rate was significantly higher than for Ca-alginate encapsulated yeasts (with 95% of confidence), and the ethanol productivity was kept above 1 g/L/h in the second recycle. However, although able to perform a third recycle, the hydrolysate still was too toxic to the cells, and fermentation stopped with only 31% of conversion (Figure 3).

**Figure 4:**
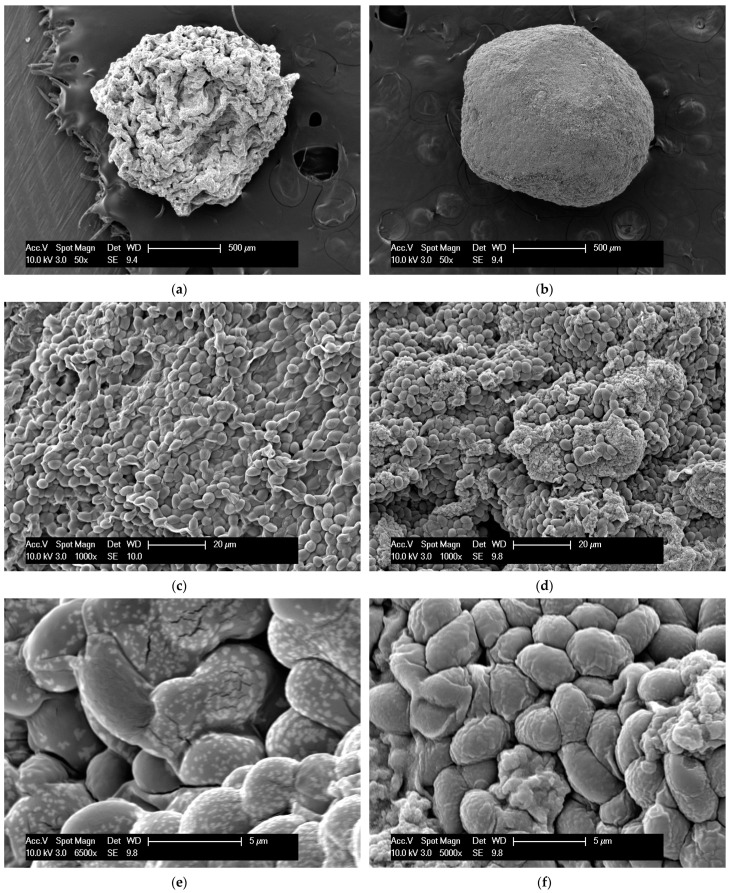
Scanning Electron Microscopy (SEM) of T18 yeast encapsulated in Ca-alginate beads (**a**,**c**,**e**) and Ca-alginate/chitosan beads (**b**,**d**,**f**).

The use of the Ca-alginate/chitosan hybrid gel led to a great improvement in the process when more stressful conditions were used, such as in the experiments using YPX with 12 g/L of acetic acid and crude hemicellulose hydrolysate. In both cases, Ca-alginate/chitosan beads showed better results. However, in less toxic environments, the profiles of fermentation of the yeasts encapsulated in the two gels were similar, which suggests that chitosan complements the protective layer for yeasts provided by alginate, producing a synergistic effect. Scanning electron microscopy (SEM) of the beads shows that the presence of chitosan in the hybrid gel really seems to grant greater structural stability to the beads (Figure 4).

**Figure 5:**
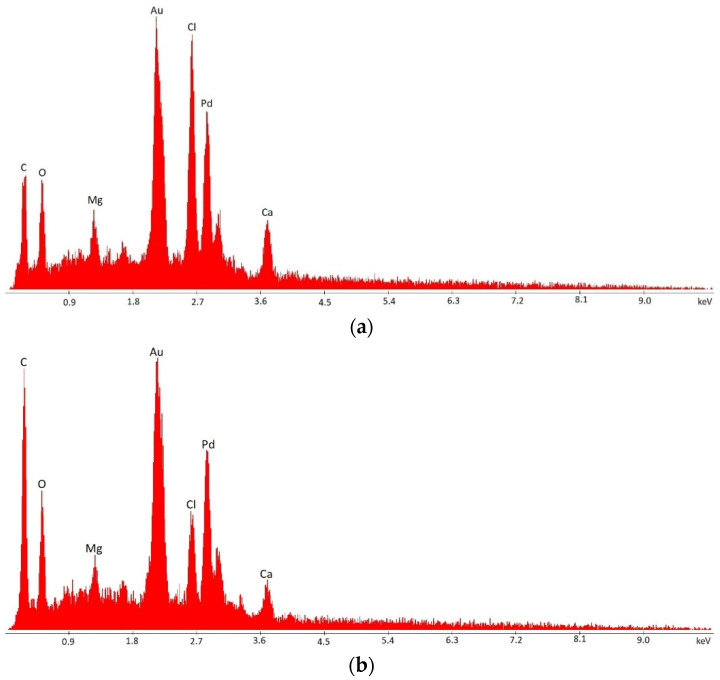
Surface composition SEM analysis of T18 yeast encapsulated in (**a**) Ca-alginate beads and (**b**) Ca-alginate/chitosan beads.

It is possible to notice that, besides structural differences, the yeasts encapsulated on Ca-alginate gel presented white spots (Figure 4e), while the same pattern was not observed in Ca-alginate/chitosan encapsulated yeasts (Figure 4f). The surface composition analysis of both gels revealed that Ca-alginate beads have a higher content of calcium, magnesium, and chlorine, probably remaining from the coagulation solution and possibly causing the observed white spots in SEM (Figure 5). Ca-alginate/chitosan beads, on the other hand, presented much a higher carbon content. This clearly shows that the chemical compositions of the two beads are different, which leads to different liquid charges in the surface area, influencing the adsorption and diffusivity of inhibitors into the beads and consequently the gradient of their concentrations and the microenvironment provided inside the beads.

## 4. Discussion

Despite the good performance of T18 yeast in the xylose fermentation, the interference of acetic acid in the ethanol productivity exposes its relevance on the homeostasis and metabolism of the T18 yeast. According to Bellissimi et al. [10], since acetic acid is a carboxylic acid with pKa 4.76, its ability to affect intracellular pH is supported by the Henderson–Hasselbach relationship. Therefore, toxicity is strongly increased at pH values below the ionization constant, as it will be largely undissociated, promoting facilitated diffusion across the membrane and cytosol acidification. Metabolically, when exposed to this condition, the yeast must activate export mechanisms against the anionic balance of intracellular acetate. This task, in search of neutrality, promotes the decoupling of energy production and nutritional transport, compromising growth and metabolism, resulting in cell death and low fermentation yield [15].

Other aromatic inhibitors mentioned above, such as furans and phenols, were not quantified in this study, but they are present in the lignocellulosic hydrolysate. These compounds, bearing different functional groups in their structures, influence the inhibitory potential, generally compromising cell growth and ethanol productivity (Qp) of the yeast *Saccharomyces cerevisiae* [35]. In addition, due to the large number of genes involved and the complexity of the stress response to inhibitors, only the biomolecular development of new and more tolerant yeasts will have limited success, since these genetic modifications could compromise other important properties in industrial yeast strains [36], necessitating the application of bioprocess design techniques to improve process feasibility (e.g., cell immobilization).

In this sense, the present work showed that the cell entrapment in Ca-alginate based gels served as an important protective barrier against crude hydrolysate toxicity, as expected [15,16]. In addition, the adsorption capacity and the binding strength are among the main factors to be considered in the choice of the appropriate immobilization material [34]. Chitosan gel is a hydrophilic polycation (pKa 6.5–7.9) which is dependent on the degree of acetylation to express its physicochemical properties. In general, it has advantages such as good mechanical strength, biocompatibility, hydrophilicity, biodegradability, bioactivity, and antimicrobial effects [37,38,39]. As for disadvantages, it has low adsorption in aqueous media. To circumvent these issues, chitosan preparation in acetic acid is adopted, which, due to the pH adjustment, interferes with the resizing of the pore diameters in the derivatization of functional groups, in addition to the chemical modification by crosslinking and grafting [40]. In this work, the crosslinker glutaraldehyde is used, which is responsible for the reactivity of the aldehyde groups with the amino groups in the formation of three-dimensional networks.

The alginate gel can be described as an anionic hydrophilic copolymer (pKa 3.5) which is easily crosslinkable, which limits the access of inhibitors with intraparticle negative charges (such as acetic acid) due to the formation of a concentration gradient. In addition, it is characterized by advantages such as high biocompatibility, biodegradability, and renewability [15,37,39]. On the other hand, mechanical strength, stability, and thermal resistance are relatively low, requiring physical or chemical modifications to increase their adsorption capacities and strengths [24]. Therefore, in this work, the use of a coagulation solution based on divalent chlorides is adopted, seeking to solidify the crosslinked adsorbency in calcium, because, under these circumstances, the reactivity resulting from the ion exchange is favored without compromising stability.

Consequently, the consortium of these biopolymers as a polyelectrolyte complex allows the union of their physicochemical properties in a synergistic way, formed by ionic gelation, in the ionization of the carboxylic group in alginate and in the protonation of the amino group in chitosan, which leads to electrostatic interactions that reduce porosity and provide protection to the encapsulated biocatalyst [41,42]. The main advantage observed in this technology is its non-toxicity, which allows repeated administration of encapsulated products, and its tenacity can be improved when the availability of functional groups is close to the stoichiometric ratio [42,43]. As a disadvantage, there is susceptibility to environmental parameters (e.g., pH and ionic strength) [41].

The application of repeated batches in the production of 2G bioethanol, using crude hemicellulosic hydrolysates of raw sugarcane bagasse, reinforces the feasibility of encapsulation, since the ease of reuse is a great advantage of this technique under industrial application. The potential of Ca-alginate encapsulated yeasts for this type of process are reported in the literature, where it is clear that yeast encapsulation in pure alginate provides robustness for the biocatalysts against hydrolysate toxicity not only by the existence of a yeast sacrificial layer but also by the microenvironment inside the beads and the gradient of concentration of inhibitors during diffusion in the immobilization gel layer [5,15]. The application of the hybrid Ca-alginate–chitosan gel proposed in this work further improved the biocatalyst protection due to higher interaction between immobilization support and the inhibitor compounds, making the adsorption of the inhibitors on the support stronger and hindering their diffusion into de biocatalyst’s beads. This behavior creates an ideal microenvironment inside the beads which preserves yeasts viability and performance. The application of hybrid alginate gels for 2G ethanol production using encapsulated yeasts is not much reported in literature. However, the results presented here strongly suggest that this approach deserves to be more explored further, demonstrating the potential to contribute to the development of a feasible 2G ethanol production from xylose. In addition, this technique has a practical impact which could be extended to other processes where stressful conditions are present, and also to other applications that involve the use of lignocellulosic hydrolysates with high inhibitor content, such as value-added chemical production in biorefineries (e.g., xylitol and muconic acid).

Finally, the production of bioethanol from the hemicellulose fraction of biomass is an interesting and attractive approach to be implemented in countries with well-stablished sugarcane mills of 1G ethanol production, such as Brazil, reducing CO_2_ emission through the 1G/2G co-production. This technology and use of the entire biomass are crucial for achieving sustainable development and a circular economy, being complementary to other technological proposed processes such as the aforementioned one from Brasken and the Steelanol process from LaanzaTech [44].

## 5. Conclusions

The development of recombinant strains together with its encapsulation builds a more robust biocatalysts mechanism, allowing the fermentation of non-detoxified lignocellulosic hydrolysates and the easy recovery of the biocatalysts. In addition, the implementation of the polymeric consortium using hybrid alginate–chitosan gel significantly improved yeast performance. In this sense, the use of hybrid alginate–chitosan gels for 2G ethanol production, after further studies for its optimization, could allow improvement in process performance, productivity, and yield.

## Figures and Tables

**Table 1:** Performance indexes of YPX (40 g/L) fermentations in the presence of different acetic acid concentrations (0–12.0 g/L) by T18 yeast immobilized on Ca-alginate and Ca-alginate/chitosan beads (1:1 V_beads_/V_medium_, 35 °C, 150 rpm, and pH 5.2). (a–i) Matching letters indicate that the means did not differ significantly, according to the Tukey test at 95% confidence level.

Immobilization Gel	Acetic Acid (g/L)	Ethanol (g/L)	Q_P_ (g/L/h)	Y_P/S_ (g/g)	Residual Xylose (g/L)
Ca-alginate beads	0.0	14.4 ± 0.0 ^a^	3.60 ± 0.01 ^c^	0.427 ± 0.066 ^g^	0.0 ± 0.0 ^h^
	4.0	15.4 ± 0.0 ^a^	1.93 ± 0.00 ^d^	0.388 ± 0.000 ^g^	0.0 ± 0.0 ^h^
	8.0	15.6 ± 0.5 ^a^	1.30 ± 0.01 ^e^	0.391 ± 0.012 ^g^	0.0 ± 0.0 ^h^
	10.0	14.2 ± 0.6 ^a^	1.19 ± 0.01 ^e^	0.356 ± 0.016 ^g^	0.0 ± 0.0 ^h^
	12.0	11.8 ± 0.8 ^b^	0.99 ± 0.07 ^f^	0.353 ± 0.023 ^g^	6.4 ± 0.1 ^i^
Ca-alginate/chitosan beads	0.0	14.5 ± 0.1 ^a^	3.61 ± 0.02 ^c^	0.424 ± 0.065 ^g^	0.0 ± 0.0 ^h^
	4.0	14.9 ± 0.7 ^a^	1.80 ± 0.09 ^d^	0.397 ± 0.004 ^g^	0.0 ± 0.0 ^h^
	8.0	14.9 ± 0.1 ^a^	1.24 ± 0.01 ^e^	0.405 ± 0.012 ^g^	0.0 ± 0.0 ^h^
	10.0	14.3 ± 0.3 ^a^	1.19 ± 0.02 ^e^	0.388 ± 0.007 ^g^	0.0 ± 0.0 ^h^
	12.0	13.6 ± 0.8 ^a^	1.13 ± 0.06 ^ef^	0.387 ± 0.015 ^g^	0.0 ± 0.0 ^h^

**Table 2:** Performance indexes for encapsulated T18 yeast recycles in sugarcane bagasse hemicellulose hydrolysate supplemented with urea (2 g/L) (35 °C, 150 rpm, and pH 5.2). (a–q) Matching letters indicate that the means did not differ significantly, according to the Tukey test at 95% confidence.

Immobilization Gel	Recycle	Ethanol (g/L)	Q_P_ (g/L/h)	Y_P/S_ (g/g)	r_S_ (g/L/h)	Cell Viability (%)	Residual Sugars (g/L)
Ca-alginate beads	1	24.8 ± 0.2 ^a^	1.03 ± 0.01 ^d^	0.436 ± 0.003 ^g^	8.2 ± 0.3 ^h^	76.8 ± 1.6 ^l^	0.0 ± 0.0 ^o^
	2	21.8 ± 0.2 ^b^	0.45 ± 0.00 ^e^	0.444 ± 0.004 ^g^	4.2 ± 0.0 ^i^	0.0 ± 0.0 ^m^	7.8 ± 0.5 ^p^
Ca-alginate/chitosan beads	1	25.0 ± 0.3 ^a^	1.04 ± 0.01 ^d^	0.439 ± 0.005 ^g^	17.0 ± 0.4 ^j^	95.6 ± 3.2 ^n^	0.0 ± 0.0 ^o^
	2	25.3 ± 0.1 ^a^	1.06 ± 0.00 ^d^	0.445 ± 0.002 ^g^	9.3 ± 0.3 ^h^	87.8 ± 4.9 ^n^	0.0 ± 0.0 ^o^
	3	7.8 ± 0.7 ^c^	0.16 ± 0.01 ^f^	0.443 ± 0.038 ^g^	1.6 ± 0.1 ^k^	0.0 ± 0.0 ^m^	39.3 ± 1.5 ^q^

## Data Availability

Data presented in this article are available from the corresponding author on reasonable request.
